# A novel circ_MACF1/miR-942-5p/TGFBR2 axis regulates the functional behaviors and drug sensitivity in gefitinib-resistant non-small cell lung cancer cells

**DOI:** 10.1186/s12890-021-01731-z

**Published:** 2022-01-07

**Authors:** Daping Fan, Yue Yang, Wei Zhang

**Affiliations:** Department of Respiratory Care, The First Affiliated Hospital of Harbin Medical University, No. 23, Post Street, Nangang District, Harbin City, 150001 Heilongjiang Province China

**Keywords:** Gefitinib resistance, NSCLC, circ_MACF1, miR-942-5p, TGFBR2

## Abstract

**Background:**

Resistance to gefitinib remains a major obstacle for the successful treatment of non-small cell lung cancer (NSCLC) with epidermal growth factor receptor (EGFR) mutations. In this paper, we studied the precise actions of circular RNA (circRNA) microtubule actin crosslinking factor 1 (circ_MACF1) in gefitinib resistance.

**Methods:**

We established gefitinib-resistant NSCLC cells (PC9/GR and A549/GR). The levels of circ_MACF1, microRNA (miR)-942-5p, and transforming growth factor beta receptor 2 (TGFBR2) were gauged by quantitative real-time PCR (qRT-PCR) or western blot. Subcellular fractionation and Ribonuclease R (RNase R) assays were done to characterize circ_MACF1. Cell survival, proliferation, colony formation, apoptosis, migration, and invasion were detected by 3-(4,5-dimethylthiazol-2-yl)-2,5-diphenyltetrazolium bromide (MTT), 5-Ethynyl-2’-Deoxyuridine (EdU), colony formation, flow cytometry, and transwell assays, respectively. Dual-luciferase reporter assays were used to verify the direct relationship between miR-942-5p and circ_MACF1 or TGFBR2. The xenograft assays were used to assess the role of circ_MACF1 in vivo.

**Results:**

Circ_MACF1 was down-regulated in A549/GR and PC9/GR cells. Overexpression of circ_MACF1 repressed proliferation, migration, invasion, and promoted apoptosis and gefitinib sensitivity of A549/GR and PC9/GR cells in vitro, as well as inhibited tumor growth under gefitinib in vivo. Circ_MACF1 directly targeted miR-942-5p, and miR-942-5p mediated the regulatory effects of circ_MACF1. TGFBR2 was identified as a direct and functional target of miR-942-5p. Circ_MACF1 modulated TGFBR2 expression through miR-942-5p.

**Conclusion:**

Our findings demonstrated that circ_MACF1 regulated cell functional behaviors and gefitinib sensitivity of A549/GR and PC9/GR cells at least partially by targeting miR-942-5p to induce TGFBR2 expression.

**Supplementary Information:**

The online version contains supplementary material available at 10.1186/s12890-021-01731-z.

## Introduction

Despite the advances in treatment strategies, lung cancer remains the most prevalent cause of cancer mortality throughout the world and approximately 85% of these are non-small cell lung cancer (NSCLC) [1, 2]. Activating mutations of epidermal growth factor receptor (EGFR) have been recognized as the second most common oncogenic factors for NSCLC development [3]. Gefitinib, one of the EGFR tyrosine kinase inhibitors (EGFR-TKIs), has been used for the treatment of advanced NSCLC patients with EGFR-activating mutations and has preferably achieved a superior survival benefit for these patients [4, 5]. Nonetheless, acquired resistance to gefitinib frequently arises [6]. Therefore, unveiling the mechanisms of gefitinib resistance is essential in improving the survival of drug-resistant NSCLC.

Circular RNAs (circRNAs) are created by the back-splicing of pre-messenger RNA and are crucial for epigenetic regulation [7]. CircRNAs can function as translational activators or repressors through binding to microRNAs (miRNAs) and sponging them [8]. Intense efforts have led to the identification of the implications of circRNAs in the pathogenesis and drug resistance of human cancers, including NSCLC [9–11]. Moreover, disordered circRNA expression has been documented in gefitinib-resistant NSCLC cell lines, hinting the involvement of circRNAs in NSCLC resistance to gefitinib [12]. For instance, Zhou et al*.* found that circ_0004015 worked as a driver in gefitinib resistance of HCC827 NSCLC cells depending on the regulation of miR-1183 [13]. Tao et al*.* reported that circ_0000567, a differentially expressed circRNA in NSCLC cells before and after gefitinib resistance, contributed to the proliferation of gefitinib-resistant NSCLC cells [14]. Nevertheless, the critical, precise roles of individual circRNAs in gefitinib resistance have remained largely undefined.

CircRNA microtubule actin crosslinking factor 1 (circ_MACF1, also called hsa_circ_0011780 according to the circRNA ID of circBase database), produced by the head-to-tail splicing of exons of MACF1 mRNA, has been identified as an anti-tumor factor in NSCLC by binding to miR-544a to induce F-Box and WD repeat domain containing 7 (FBXW7) [15]. In a preliminary survey for the differently expressed circRNAs in gefitinib-resistant NSCLC cells, we found that circ_MACF1 was the most significantly altered circRNA in gefitinib-resistant A549 cells (Additional file 1: Fig. S1A). However, no reports showed whether circ_MACF1 participates in gefitinib resistance in NSCLC. In this paper, we established an important role of circ_MACF1 in affecting the functional behaviors and drug sensitivity of gefitinib-resistant NSCLC cell lines. Furthermore, we uncovered a novel circ_RNA/miRNA/mRNA network in regulating gefitinib resistance of NSCLC. Our study demonstrated an epigenetic cause of gefitinib resistance with diagnostic and therapeutic implications.

**Fig. 1:**
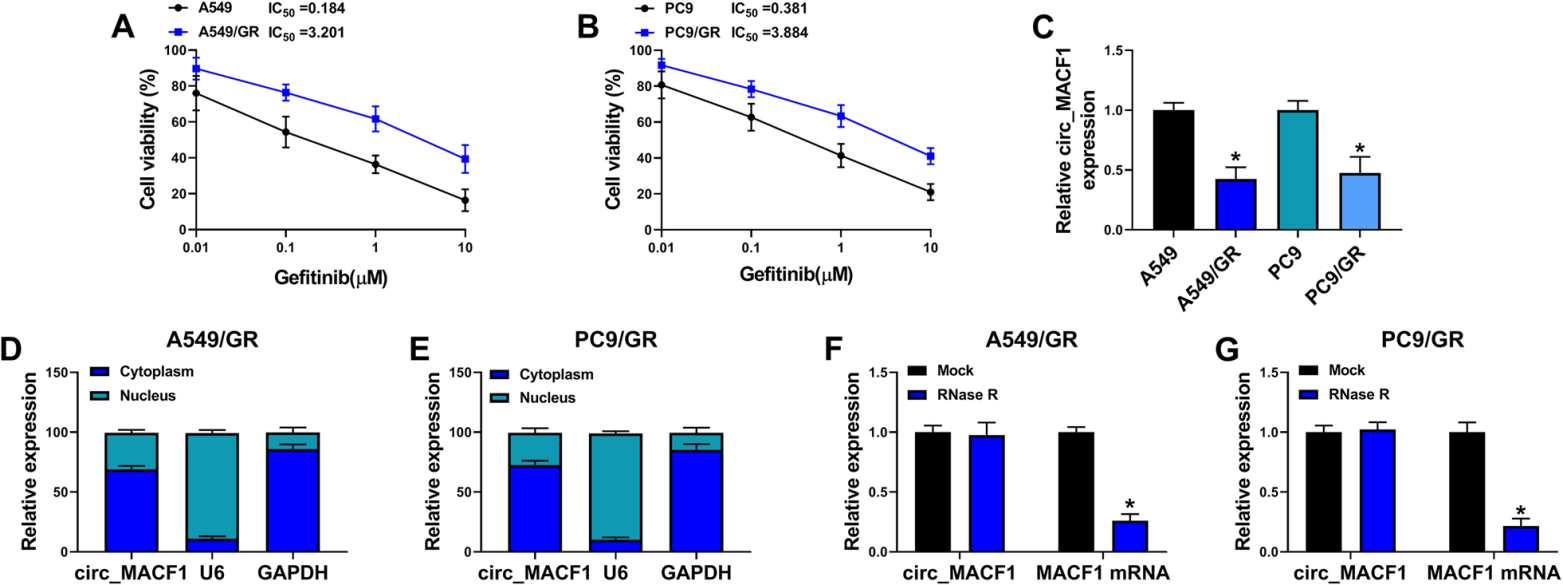
Circ_MACF1 expression is decreased in A549/GR and PC9/GR cells. **A**, **B** MTT assay of cell viability and the IC_50_ values for gefitinib in A549/GR, A549, PC9/GR, and PC9 cells. **C** qRT-PCR analysis of circ_MACF1 expression in A549/GR, A549, PC9/GR, and PC9 cells. **D**, **E** Subcellular fractionation assay showing the subcellular localization of circ_MACF1 in A549/GR and PC9/GR cells. **F**, **G** RNase R assay showing the RNase R resistance of circ_MACF1 in A549/GR and PC9/GR cells. *P < 0.05

## Materials and methods

### Human plasma samples

In this study, we enrolled a total of 31 NSCLC patients harboring EGFR-activating mutations (L858R or Ex19 deletion) who were treated with gefitinib for therapy from The First Affiliated Hospital of Harbin Medical University. Pre-gefitinib treated plasma samples were collected from these patients (defined gefitinib-sensitive/Tumor-responsive). Of these, 11 patients developed gefitinib resistance according to the Computerized Tomographic Scanning (showing tumor re-growth). Post-gefitinib treated plasma samples were collected from the 11 gefitinib-resistant patients (defined gefitinib-resistant/Tumor-resistant). The 11 pairs of pre- and post-gefitinib treated plasma samples from these resistant patients were used to measure circ_MACF1 and miR-942-5p expression by quantitative real-time PCR (qRT-PCR). The study was approved by the Ethics Committee of The First Affiliated Hospital of Harbin Medical University with written informed consent provided by all subjects.

### Cell lines

Human PC-9 (EGFR mutation-positive, exon 19 deletion) NSCLC cells were from Riken BioResource Research Center (Tokyo, Japan) and A549 (wild-type EGFR) NSCLC cells and HEK293 cells were from the American Type Culture Collection (ATCC, Manassas, VA, USA). PC9 and A549 cells were propagated in RPMI-1640 medium containing 10% fetal calf serum, and 1% antibodies (all from PAA-Laboratories, Cölbe, Germany). HEK293 cells were cultivated under standard protocols provided by ATCC. All cells were maintained at 37 °C, 85% humidity, and 5% CO_2_. We established gefitinib-resistant NSCLC cells (PC9/GR and A549/GR) by exposing PC9 and A549 cells to increasing concentrations of gefitinib (Selleck Chemicals, Houston, TX, USA) as reported [16] for 6 months.

### Lentivirus transduction and transient transfection of cells

Human hsa_circ_0011780 sequence and a scrambled control sequence, synthesized by BGI (Shenzhen, China), were individually inserted into the CircRNA expression lentiviral vector pLO5-ciR (Geneseed) to produce circ_MACF1 overexpression plasmid and negative control Vector plasmid. Lentiviruses expressing circ_MACF1 (lenti-circ_MACF1) or a scrambled control (lenti-NC) were generated by co-transfection of the corresponding plasmid construct and psPAX2 and pMD2.G packaging vectors (Addgene, Cambridge, MA, USA) into HEK293 cells. Virus was harvested after 48 h, and A549/GR cells were incubated with viral supernatants containing 8 µg/mL polybrene (Sigma-Aldrich, Seelze, Germany). Following a 24-h incubation period, puromycin (Sigma-Aldrich) was added into the media at 2 µg/mL, and virus-positive infected cells were selected for 7 days.

For transient transfection, PC9/GR and A549/GR cells (1 × 10^5^) were plated in 24-well culture dishes 18 h before transfection. The following day, 200 ng of plasmid construct, 30 nM of miRNA mimic (miR-NC or miR-942-5p), 50 nM of miRNA inhibitor (anti-miR-NC or anti-miR-942-5p), or 80 nM of siRNA (si-NC or si-TGFBR2 designed for transforming growth factor beta receptor 2 (TGFBR2) depletion), was introduced into the cells using Lipofectamine 3000 as recommended by the manufacturers (Invitrogen, Cergy Pontoise, France). We harvested the transfected cells for in vitro assays after culturing for 24 h. All oligonucleotides were from Ribobio (Guangzhou, China) and their sequences were described in Additional file 3: Table S1.

### RNA preparation and qRT-PCR

We prepared total RNA from cultured cells and tissue samples with peqGOLD total RNA Kit (PeqLab, Erlangen, Germany) and quantified it using Agilent 2100 Bioanalyzer (Agilent Technologies, Stockport, UK). For analysis of circ_MACF1 and mRNA, cDNA was randomly or oligo(dT) primed from 1–2 µg of total RNA using ReverTra Ace RT Kit as per the manufacturing instructions (Toyobo, Tokyo, Japan). For analysis of miR-942-5p, 200 ng of RNA was conversed to cDNA using BON-miR miRNA 1st-strand cDNA Synthesis Kit (Bonyakhteh, Tehran, Iran) based on the accompanying recommendations. Synthesized cDNA was amplified by qRT-PCR using Thunderbird SYBR® qPCR Mix (Toyobo) with specific primers (Additional file 3: Table S1) on the MyiQ Detection System (Bio-Rad, Gladesville, NSW, Australia). Fold changes were expressed as a corrected value obtained by dividing the expression level by that for β-actin or U6 and calculated under usage of the 2^−ΔΔCt^ method [17].

### Subcellular fractionation assay

Preparation of cytoplasmic and nuclear RNA of PC9/GR and A549/GR cells was carried out under usage of the Cytoplasmic & Nuclear RNA Purification Kit as per the manufacturing guidance (Norgen Biotek, Thorold, ON, Canada). The levels of circ_MACF1, U6 (nuclear control), and glyceraldehyde-3-phosphate dehydrogenase (GAPDH, cytoplasmic control) were gauged by qRT-PCR as above.

### Ribonuclease R (RNase R) assay

Total RNA (2.5 µg) was incubated with 10 units of RNase R for 15 min at 37 °C as described by the manufacturers (Geneseed). We examined the levels of circ_MACF1 and the corresponding linear MACF1 mRNA by qRT-PCR as above.

### Evaluation of cell survival and proliferation

We utilized the 3-(4,5-dimethylthiazol-2-yl)-2,5-diphenyltetrazolium bromide (MTT) assay to evaluate cell survival and proliferation. For cell survival, cultured cells were seeded in 96-well dishes at 5,000 cells per well and the gefitinib (0.01, 0.05, 0.1, 0.5, 1, 5, and 10 µM) were added the following day. After a 24-h incubation period, the number of living cells was assessed by MTT assay under standard protocols [18]. The half maximal inhibitory concentration (IC_50_ value) was determined following curve fitting to the cell survival data. For cell proliferation, transfected PC9/GR and A549/GR cells (2,000 cells/well) were plated in 96-well dishes in growth media and maintained at 37 °C. After desired duration of culture (1, 2, and 3 days), MTT solution and DMSO were used as per the manufacturing guidance (Solarbio, Beijing, China). The optical density at 570 nm was detected using a PowerWave340™ reader (BioTek Instruments, Winooski, VT, USA).

### 5-ethynyl-2’-deoxyuridine (EdU) assay

Evaluation of cell proliferation was conducted under usage of a Cell-Light EdU Apollo488 In Vitro Kit as recommended by the manufacturers (Ribobio). Transfected PC9/GR and A549/GR cells (1 × 10^4^ cells/well) grown in 96-well dishes were incubated with EdU solution (50 µM). Following a 2-h incubation period, cells were stained with 1 × Apollo488 solution for 30 min and 1 × Hoechst 33342 for 30 min (nuclei staining). The cells were visualized by the DM2500 fluorescence microscope (Leica, Wetzlar, Germany). The proliferation rate of cells was determined by calculating the ratio of EdU-positive nuclei (green) to total nuclei (blue).

### Cell colony formation assay

We plated transfected PC9/GR and A549/GR cells at a clonal density (~ 100 cells per well) into 6-well dishes. Twelve days later, the wells were stained with 0.1% crystal violet (Solarbio), and the colonies (> 50 cells) were scored in 10 randomly selected fields each sample under an Image J-software (National Institutes of Health, Bethesda, MD, USA).

### Flow cytometry for cell apoptosis

Approximately 1 × 10^6^ transfected PC9/GR and A549/GR cells each sample were collected, pelleted, and resuspended in incubation buffer containing propidium iodide (PI, 50 µg/mL) and Annexin V-fluorescein isothiocyanate (FITC) based on the instructions of the Detection Kit (BD Biosciences, Heidelberg, Germany). For data analysis, we applied an LSRII cytometer with FACSDiva software from BD Biosciences.

### Transwell migration and invasion assays

For invasion analysis, transfected PC9/GR and A549/GR cells (1 × 10^5^ cells/well) in serum-free media were seeded on the top of 24-well inserts pre-coating with Matrigel (pore size, 8 µm; Millipore, Shanghai, China). For migration analysis, transfected PC9/GR and A549/GR cells (3 × 10^4^ cells/well) were seeded on the top of non-coated insert membranes (Millipore). The 24-well inserts were placed in 24-well dishes containing growth media. Following a 24-h incubation period, the cells on the lower surface of the inserts were stained with 0.1% crystal violet, photographed under a 100 × invert microscope (Leica), and counted under the Image J-software.

### Western blot

We carried out western blot under standard methods [15]. We used the following primary antibodies: rabbit anti-Twist1 antibody (#69366; dilution 1:1,000) from Cell Signaling Technology (Danvers, MA, USA), mouse E-cadherin antibody (#14472; dilution 1:1,000) from Cell Signaling Technology, rabbit anti-TGFBR2 antibody (ab186838; dilution 1:1,000) from Abcam (Cambridge, UK), and rabbit anti-GAPDH loading control (#5174; dilution 1:2,000) from Cell Signaling Technology. The nitrocellulose-bound primary antibodies were incubated with goat anti-mouse or anti-rabbit IgG secondary antibody labeled by horseradish peroxidase (ab6789 or ab97051; dilution 1:10,000) from Abcam and detected by the Dura Detection Kit (Perbio Sciences, Bonn, Germany). Data acquisition was performed using the Chemidoc-XRS gel documentation system with Quantity One software as recommended by the manufacturers (Bio-Rad).

### Bioinformatics and dual-luciferase reporter assay

We searched the miRNA-binding sites to circ_MACF1 and human 3’untranslated region (3’UTR) by circInteractome web (https://circinteractome.nia.nih.gov/index.html) and ENCORI prediction software (http://starbase.sysu.edu.cn/), respectively. The fragments of circ_MACF1 and TGFBR2 3’UTR harboring the miR-942-5p pairing sites or miss-matched target sequence, provided by BGI, were individually subcloned into the psiCHECK-2 vector (Promega, Leiden, The Netherlands). The appropriate reporter construct (200 ng) was transfected into PC9/GR and A549/GR cells (1 × 10^5^) using Lipofectamine 3000 together with miRNA mimic at 30 nM. After a 48-h transfection period, we analyzed the luciferase activity with Dual-Glow Luciferase Assay System from Promega.

### RNA pull-down assay

We obtained biotinylated circ_MACF1 probe and negative Oligo probe from Ribobio. Cultured A549/GR cells were lysed in RIPA lysis buffer. Then, lysates were incubated with circ_MACF1 probe or Oligo probe and Streptavidin beads (Invitrogen) overnight at 4 °C. RNA bound to beads was measured by qRT-PCR to evaluate circ_MACF1 and miRNAs levels.

### In vivo tumor growth in xenograft model

Twelve athymic BALB/c female nude mice aged 6–8 weeks (Vital River Laboratory Animal Technology Co., Ltd., Beijing, China) were used for the experiments in accordance with a protocol approved by the Animal Care and Use Ethics Committee of The First Affiliated Hospital of Harbin Medical University. The tumorigenicities of the A549/GR cells transduced by lenti-circ_MACF1 or lenti-NC were assayed by the subcutaneous implantation of 1 × 10^6^ cells in 150 µL PBS into the right flanks of BALB/c mice (6 mice per group), with gefitinib administration (30 mg/kg) per os 10 days after implantation. In the meantime, tumor size was gauged every 5 days using Vernier Calipers. Tumor volume measurement was done under usage of the formula (length × width^2^/2). All mice were euthanized on day 30rd with CO_2_ overdose, and the xenograft tumors were removed, weighed, and further analyzed. The tumors embedded in paraffin were subjected to immunohistochemistry assays using rabbit anti-TGFBR2 antibody (PA5-88257; dilution 1:100) from Invitrogen and rabbit anti-Ki67 antibody (ab16667; dilution 1:200) from Abcam, as described elsewhere [19].

### Statistical analysis

Data were presented as means of at least 3 independent experiments, with standard deviation of mean error bars. Data sets were compared with the paired or unpaired Student’s *t*-test or analysis of variance with Dunnett’s multiple comparison test, where appropriate. Differences were considered significant when *P* < 0.05.

## Results

### Circ_MACF1 is associated with gefitinib resistance of NSCLC

To preliminarily evaluate the resistance mechanisms in NSCLC, we firstly established two gefitinib-resistant NSCLC cell lines (A549/GR and PC9/GR) by prolonged exposure to gefitinib. MTT assay confirmed the successful establishment of gefitinib-resistant NSCLC cells, as presented by the increased IC_50_ value for gefitinib in A549/GR and PC9/GR cells as compared with that in their parental cells (Fig. 1A, B). Interestingly, analysis of circ_MACF1 expression in the two gefitinib-resistant cell lines showed that circ_MACF1 was markedly inhibited in A549/GR and PC9/GR cells compared with the corresponding sensitive cells (Fig. 1C). In contrast to the pre-gefitinib treated plasma, circ_MACF1 was highly down-regulated in gefitinib-resistant NSCLC plasma (Additional file 2: Fig. S2A). To elucidate the subcellular localization of circ_MACF1 in A549/GR and PC9/GR cells, we performed subcellular fractionation assays. Circ_MACF1 was mainly present in the cytoplasm (Fig. 1D, E). Additionally, incubation of cellular RNA with RNase R resulted in a striking reduction in the levels of the corresponding MACF1 linear mRNA, and circ_MACF1 was stable and resistant to RNase R (Fig. 1F, G). Together, these data suggest that down-regulation of circ_MACF1 may be associated with gefitinib resistance of NSCLC.

**Fig. 2:**
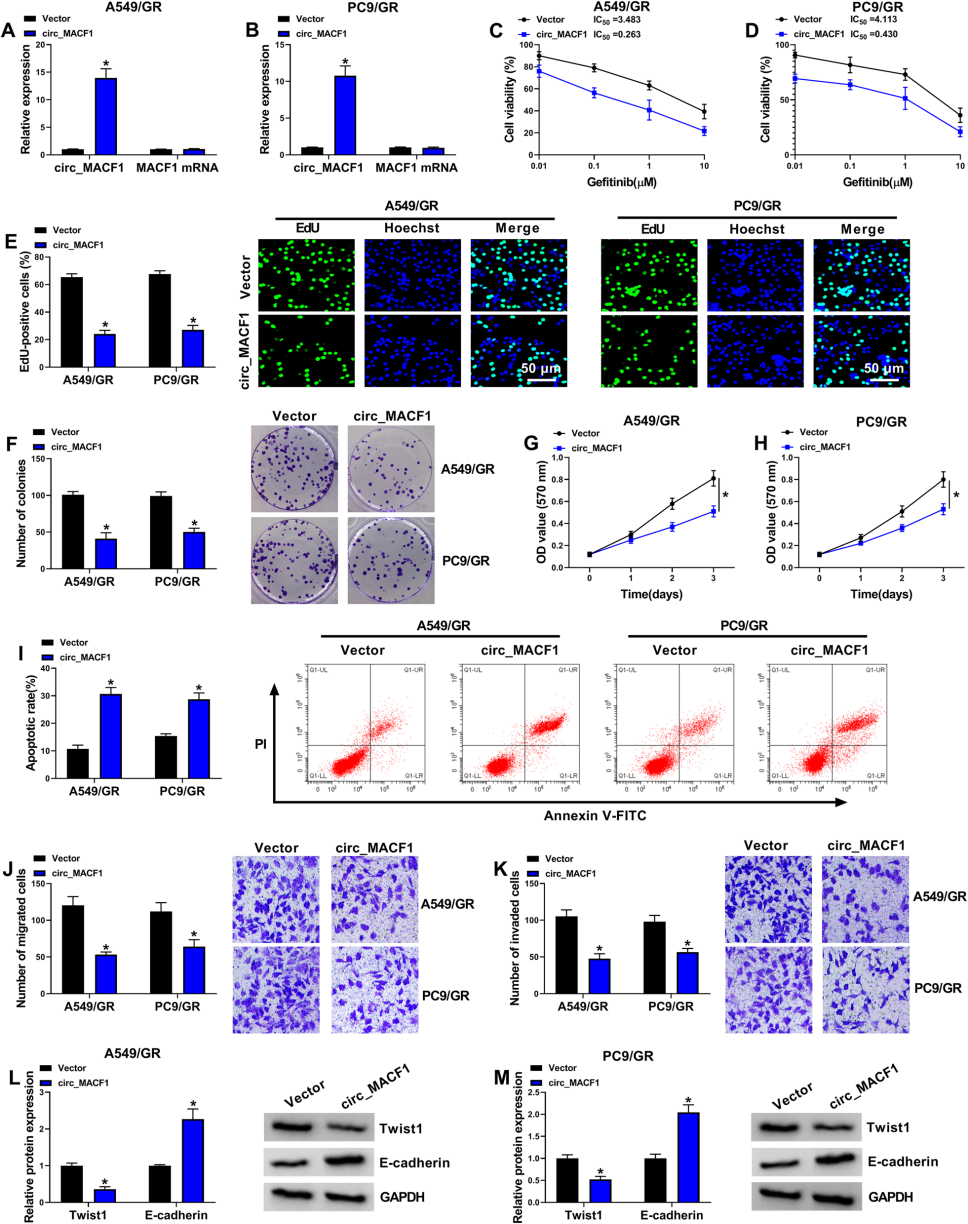
Circ_MACF1 affects the functional behaviors and gefitinib sensitivity of A549/GR and PC9/GR cells. A549/GR and PC9/GR cells were transfected with negative control Vector plasmid or circ_MACF1 overexpression plasmid. **A**, **B** qRT-PCR analysis of circ_MACF1 and MACF1 mRNA in transfected A549/GR and PC9/GR cells. **C**, **D** MTT assay of cell viability and the IC_50_ values for gefitinib in transfected A549/GR and PC9/GR cells. **E** Representative images depicting a cell proliferation assay and cell proliferation by EdU assay. **F** Representative images showing a cell colony formation assay in transfected A549/GR and PC9/GR cells. **G**, **H** MTT assay for proliferation ability of transfected A549/GR and PC9/GR cells. **I** Representative images describing a cell apoptosis assay and flow cytometry for cell apoptosis. **J**, **K** Representative images depicting cell migration and invasion assays performed by flow cytometry. **L**, **M** Western blot showing Twist1 and E-cadherin levels in transfected A549/GR and PC9/GR cells. *P < 0.05

### Overexpression of circ_MACF1 represses proliferation, migration, invasion, and promotes apoptosis and gefitinib sensitivity of A549/GR and PC9/GR cells

To directly elucidate the functional role of circ_MACF1 in gefitinib resistance of NSCLC, we performed gain-of-function experiments in A549/GR and PC9/GR cells. In comparison to the Vector controls, transfection of circ_MACF1 overexpression plasmid remarkably up-regulated circ_MACF1 expression but not affected the corresponding MACF1 linear mRNA (Fig. 2A, B). Notably, overexpression of circ_MACF1 decreased the IC_50_ value for gefitinib in A549/GR and PC9/GR cells (Fig. 2C, D), indicating that circ_MACF1 overexpression sensitized gefitinib-resistant NSCLC cells to gefitinib. Moreover, enforced expression of circ_MACF1 strongly impeded cell proliferation, colony formation (Fig. 2E–H), and enhanced cell apoptosis (Fig. 2I), as well as hindered cell migration and invasion (Fig. 2J, K). Additionally, overexpression of circ_MACF1 led to a clear decrease in the expression of Twist1 and a distinct increase in the level of E-cadherin in the two gefitinib-resistant NSCLC cell lines (Fig. 2L, M). These results collectively demonstrate that circ_MACF1 can regulate the functional behaviors and gefitinib sensitivity of gefitinib-resistant NSCLC cell lines.

### Circ_MACF1 directly targets miR-942-5p

**Fig. 3:**
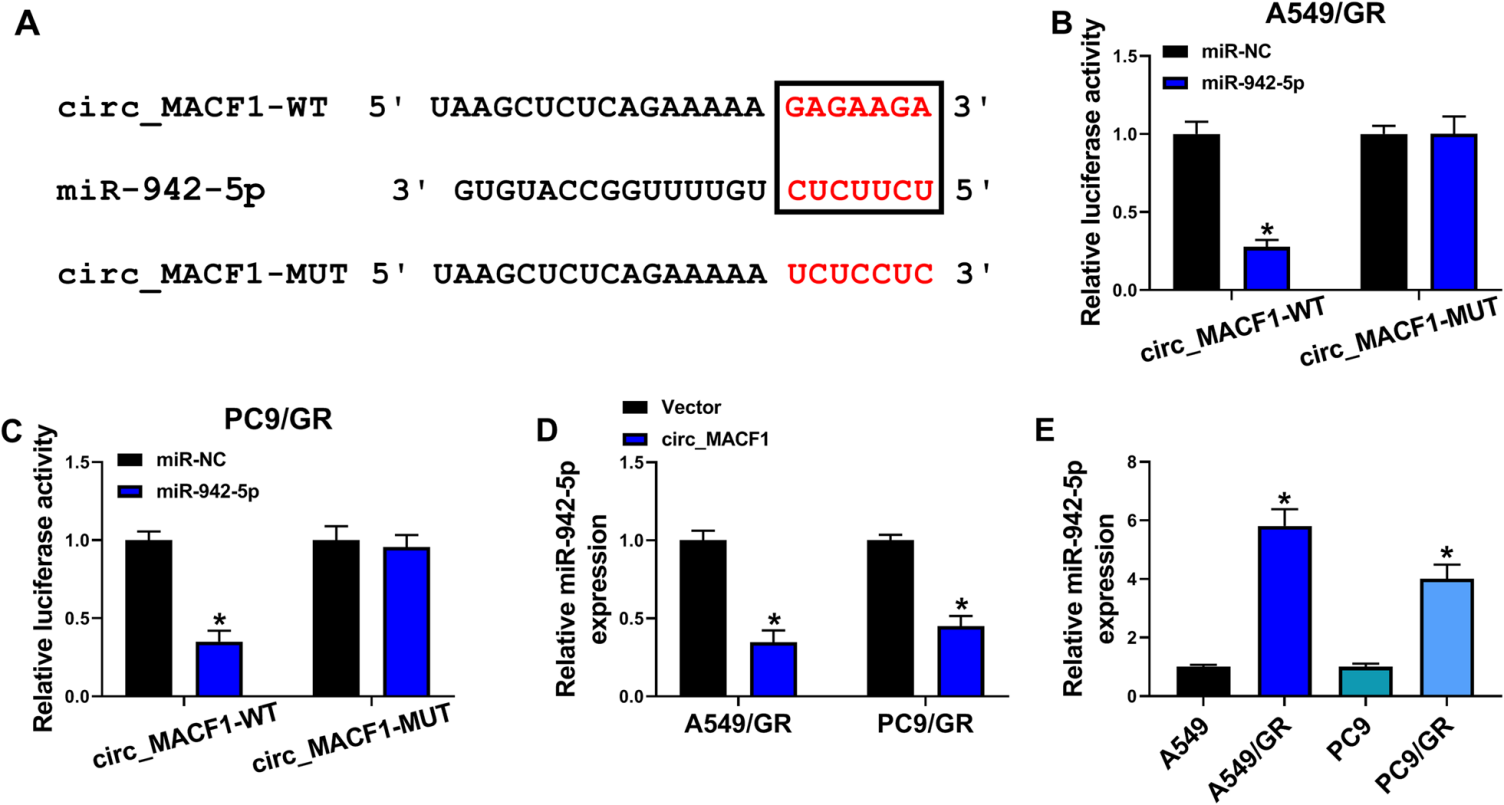
Circ_MACF1 directly targets miR-942-5p. **A** Sequence of miR-942-5p, the putative complementary sequence for miR-942-5p within circ_MACF1, and the mutation in the miR-942-5p target region. **B**, **C** Dual-luciferase reporter assays in A549/GR and PC9/GR cells co-transfected with miR-942-5p mimic or miR-NC control mimic and circ_MACF1 wild-type (circ_MACF1-WT) or mutant-type (circ_MACF1-MUT) luciferase reporters. **D** qRT-PCR analysis showing miR-942-5p expression in A549/GR and PC9/GR cells transfected by negative control Vector plasmid or circ_MACF1 overexpression plasmid. **E** qRT-PCR analysis of miR-942-5p expression in A549/GR, A549, PC9/GR, and PC9 cells. *P < 0.05

To further understand the role of circ_MACF1, we carried out a detailed analysis of the miRNAs that potentially bind to circ_MACF1. Among these candidates predicted by circInteractome web, we selected several miRNAs that were associated with NSCLC pathogenesis and found that miR-942-5p abundance was significantly elevated by biotinylated circ_MACF1 probe in A549/GR cells (Additional file 1: Fig. S1B and S1C). In database prediction by circInteractome web of the targeted miRNAs of circ_MACF1 revealed that circ_MACF1 harbors a putative complementary sequence for miR-942-5p (Fig. 3A). To confirm whether the complementary sites are validity for the direct relationship between circ_MACF1 and miR-942-5p, we constructed circ_MACF1 wild-type (circ_MACF1-WT) or mutant-type (circ_MACF1-MUT) luciferase reporters and assayed them in luciferase assays. Transfection of circ_MACF1-WT in the presence of miR-942-5p mimic produced a striking reduction of relative luciferase activity (about 72% in A549/GR cells and 65% in PC9/GR cells), and this effect was abrogated by circ_MACF1-MUT carrying a mutated binding region (Fig. 3B, C). Moreover, we observed a remarkable down-regulation in the expression of endogenous miR-942-5p in circ_MACF1-overexpressing A549/GR and PC9/GR cells (Fig. 3D). Additionally, qRT-PCR analysis showed that gefitinib-resistant NSCLC plasma exhibited higher levels of miR-942-5p compared with gefitinib-sensitive controls (Additional file 2: Fig. S2B). In line with tissue samples, miR-942-5p expression was elevated in A549/GR and PC9/GR cells compared with the corresponding sensitive cells (Fig. 3E). All these data strongly establish the notion that circ_MACF1 directly targets miR-942-5p and represses its expression.

### MiR-942-5p is a molecular mediator of circ_MACF1 function in A549/GR and PC9/GR cells

**Fig. 4:**
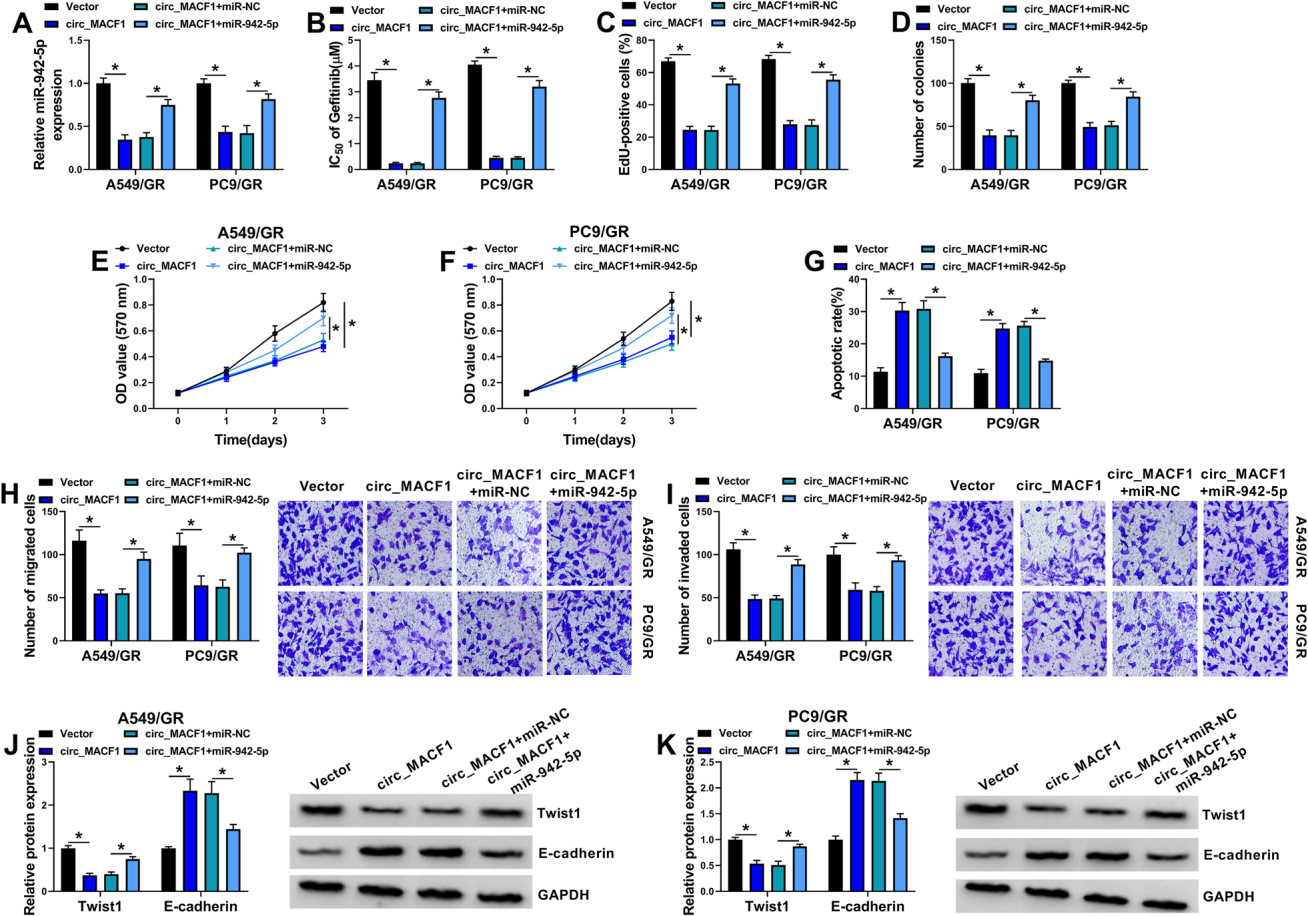
Circ_MACF1 overexpression affects the functional behaviors and gefitinib sensitivity of A549/GR and PC9/GR cells by down-regulating miR-942-5p. A549/GR and PC9/GR cells were transfected with negative control Vector plasmid, circ_MACF1 overexpression plasmid, circ_MACF1 overexpression plasmid + miR-NC mimic, or circ_MACF1 overexpression plasmid + miR-942-5p mimic. **A** Relative miR-942-5p expression by qRT-PCR analysis in transfected A549/GR and PC9/GR cells. **B** MTT assay for the IC_50_ value for gefitinib in transfected A549/GR and PC9/GR cells. **C** Cell proliferation by EdU assay. **D** Cell colony formation by colony formation assay. **E**, **F** MTT assay for cell proliferation. **G** Apoptosis of transfected A549/GR and PC9/GR cells by flow cytometry. **H**, **I** Transwell assay for cell migration and invasion abilities in transfected A549/GR and PC9/GR cells. **J**, **K** Western blot showing the expression levels of Twist1 and E-cadherin in transfected A549/GR and PC9/GR cells. *P < 0.05

We next asked whether circ_MACF1 exerted regulatory effects in the functional behaviors of gefitinib-resistant NSCLC cells by miR-942-5p. To address this, we overexpressed miR-942-5p by miR-942-5p mimic introduction in circ_MACF1-overexpressing A549/GR and PC9/GR cells. The effectiveness of miR-942-5p mimic in increasing miR-942-5p level was confirmed by qRT-PCR (Fig. 4A). By contrast, miR-942-5p expression restoration remarkably reversed reduction of the IC_50_ value for gefitnib of circ_MACF1 overexpression in the two gefitinib-resistant cell lines (Fig. 4B). Moreover, restored expression of miR-942-5p abolished circ_MACF1 overexpression-driven anti-proliferation, anti-colony formation (Fig. 4C–F), pro-apoptosis (Fig. 4G), anti-migration (Fig. 4H), and anti-invasion (Fig. 4I) effects. Additionally, miR-942-5p level restoration counteracted Twist 1 and E-cadherin expression alteration induced by circ_MACF1 overexpression in A549/GR and PC9/GR cells (Fig. 4J, K). Taken together, these data suggest that the effects of circ_MACF1 overexpression in A549/GR and PC9/GR cells may be partially due to down-regulation of miR-942-5p.

### Circ_MACF1 regulates TGFBR2 expression by targeting miR-942-5p

**Fig. 5:**
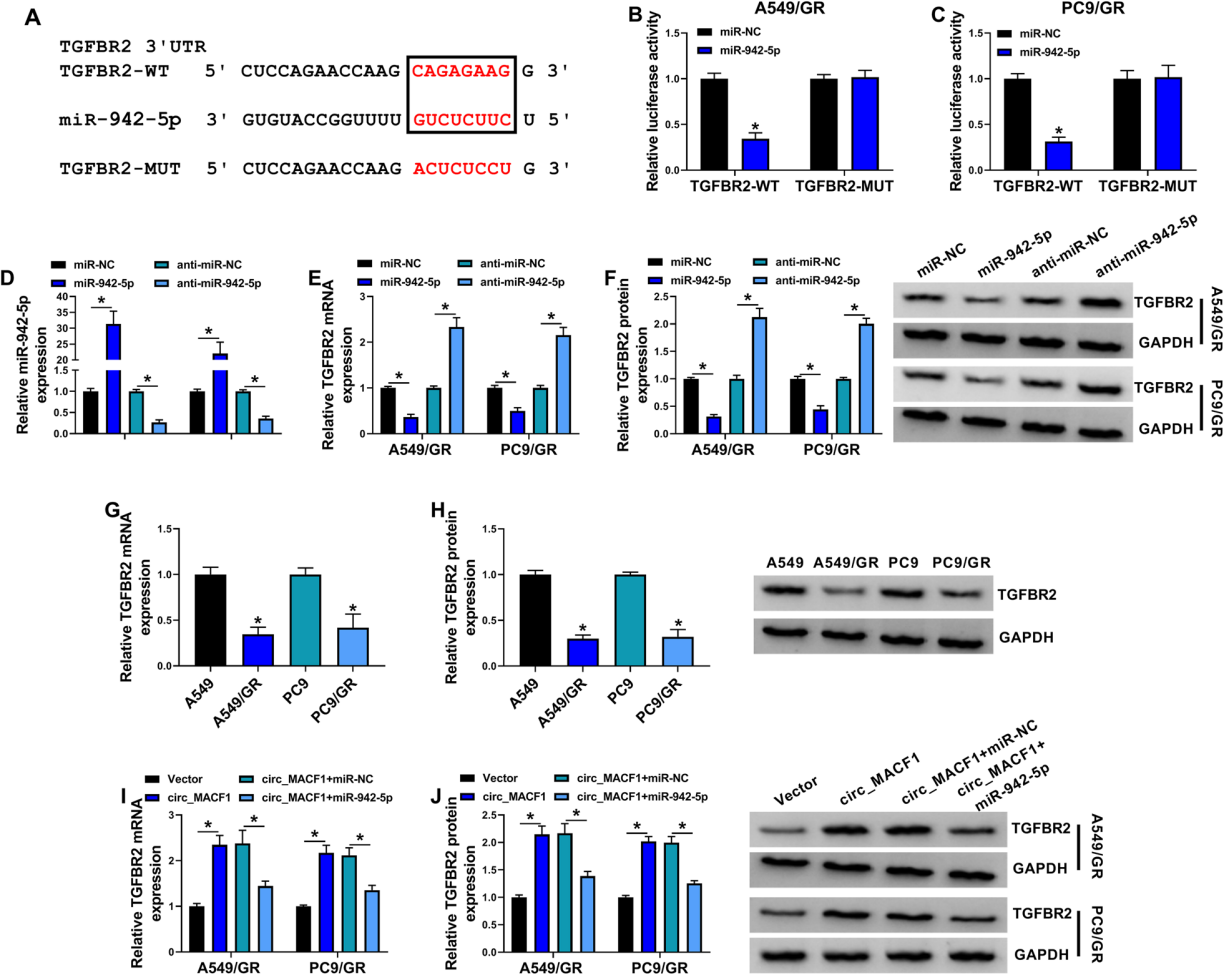
Circ_MACF1 targets miR-942-5p to regulate TGFBR2 expression. **A** Sequence of miR-942-5p, the putative complementary region for miR-942-5p within TGFBR2 3’UTR, and the mutation in the seed region of the miR-942-5p target sequence. **B**, **C** Dual-luciferase reporter assays in A549/GR and PC9/GR cells co-transfected with miR-942-5p mimic or miR-NC control mimic and TGFBR2 3’UTR wild-type (TGFBR2-WT) or mutant-type (TGFBR2-MUT) luciferase reporters. Relative miR-942-5p expression by qRT-PCR analysis (**D**), TGFBR2 mRNA level by qRT-PCR analysis (**E**), TGFBR2 protein level by western blot (**F**), in A549/GR and PC9/GR cells transfected by miR-NC mimic, miR-942-5p mimic, anti-miR-NC, or anti-miR-942-5p. TGFBR2 mRNA level by qRT-PCR analysis and TGFBR2 protein level by western blot in A549/GR, A549, PC9/GR, and PC9 cells (**G**, **H**), A549/GR and PC9/GR cells transfected by negative control Vector plasmid, circ_MACF1 overexpression plasmid, circ_MACF1 overexpression plasmid + miR-NC mimic, or circ_MACF1 overexpression plasmid + miR-942-5p mimic (**I**, **J**). *P < 0.05

To identify the downstream effectors of miR-942-5p, we used target-prediction ENCORI software. Among these candidates, we selected several genes that were involved in NSCLC progression, and we observed a striking elevation in the level of TGFBR2 mRNA in miR-942-5p-silenced A549/GR cells (Additional file 1: Fig. S1D). Computational prediction of human miR-942-5p targets by ENCORI software shows a putative complementary sequence for miR-942-5p within TGFBR2 3’UTR (Fig. 5A). Transfection of miR-942-5p mimic, but not the miR-NC control, markedly down-regulated the luciferase activity of TGFBR2 3’UTR luciferase reporter (TGFBR2-WT, Fig. 5B, C). However, this reduction was abolished by a 3’UTR reporter (TGFBR2-MUT) that contained specific point mutations in the seed region of the miR-942-5p target sequence (Fig. 5B, C). To experimentally support that miR-942-5p can target TGFBR2, we analyzed TGFBR2 expression in A549/GR and PC9/GR cells after alteration of miR-942-5p. The effectiveness of miR-942-5p mimic and inhibitor (anti-miR-942-5p) in elevating and repressing miR-942-5p was validated by qRT-PCR (Fig. 5D). By contrast, A549/GR and PC9/GR cells overexpressing miR-942-5p exhibited lower levels of TGFBR2 mRNA and protein, and we observed a clear elevation in the levels of TGFBR2 mRNA and protein in miR-942-5p-silencing cells (Fig. 5E, F). Consistent with circ_MACF1 expression, A549/GR and PC9/GR cells showed lower levels of TGFBR2 mRNA and protein compared with their sensitive parents (Fig. 5G, H).

On the basis of the above observations, we decided to elucidate if circ_MACF1 could modulate TGFBR2 expression through miR-942-5p. We transfected circ_MACF1 overexpression plasmid alone or together with miR-942-5p mimic into A549/GR and PC9/GR cells and checked for TGFBR2 expression. As would be expected, circ_MACF1 overexpression led to a strong elevation in the levels of TGFBR2 mRNA and protein in A549/GR and PC9/GR cells, and this effect was abrogated by miR-942-5p up-regulation (Fig. 5I, J). All these results point to the regulation of circ_MACF1 on TGFBR2 expression through miR-942-5p.

### TGFBR2 is a downstream effector of the circ_MACF1/miR-942-5p axis in affecting the functional behaviors and gefitinib sensitivity of A549/GR and PC9/GR cells

**Fig. 6:**
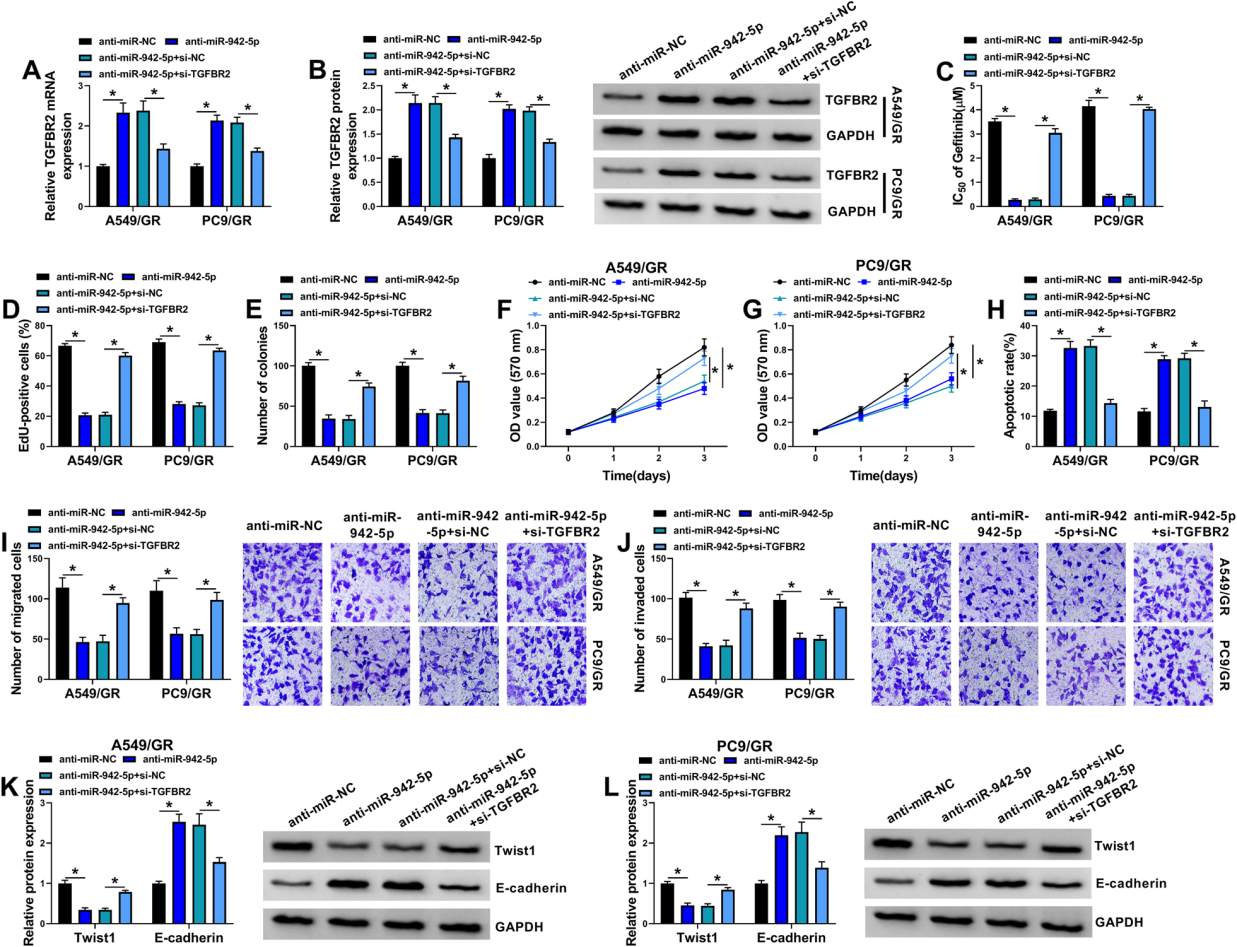
Knockdown of miR-942-5p modulates the functional behaviors and gefitinib sensitivity of A549/GR and PC9/GR cells by up-regulating TGFBR2. A549/GR and PC9/GR cells were transfected with anti-miR-NC, anti-miR-942-5p, anti-miR-942-5p + si-NC, or anti-miR-942-5p + si-TGFBR2 and checked for TGFBR2 mRNA expression by qRT-PCR analysis (**A**), TGFBR2 protein level by western blot (**B**), IC_50_ value for gefitinib by MTT assay (**C**), cell proliferation by EdU assay (**D**), cell colony formation by colony formation assay (**E**), cell proliferation by MTT assay (**F**, **G**), cell apoptosis by flow cytometry (**H**), cell migration and invasion by transwell assay (**I**, **J**), Twist1 and E-cadherin levels by western blot (**K**, **L**). *P < 0.05

Having established that miR-942-5p directly targets TGFBR2, we then undertook to examine if TGFBR2 represents a functionally downstream effector of miR-942-5p in affecting cell functional behaviors. To address this possibility, we reduced TGFBR2 expression with si-TGFBR2 in miR-942-5p-silenced A549/GR and PC9/GR cells. The transfection efficiency of TGFBR2 targeting siRNA (si-TGFBR2) was examined by qRT-PCR and western blot (Fig. 6A, B). In contrast to the anti-miR-NC control, reduced expression of miR-942-5p markedly inhibited the IC_50_ value for gefitinib (Fig. 6C), suppressed cell proliferation, colony formation (Fig. 6D–G), and enhanced cell apoptosis (Fig. 6H), as well as diminished cell migration (Fig. 6I), and invasion (Fig. 6J). Also, knockdown of miR-942-5p decreased Twist1 expression and up-regulated E-cadherin level in A549/GR and PC9/GR cells (Fig. 6K, L). However, these effects of miR-942-5p depletion were significantly abolished by TGFBR2 down-regulation (Fig. 6C–L). Together, these data suggest that the observed effects of miR-942-5p may be due to up-regulation of TGFBR2.

**Fig. 7:**
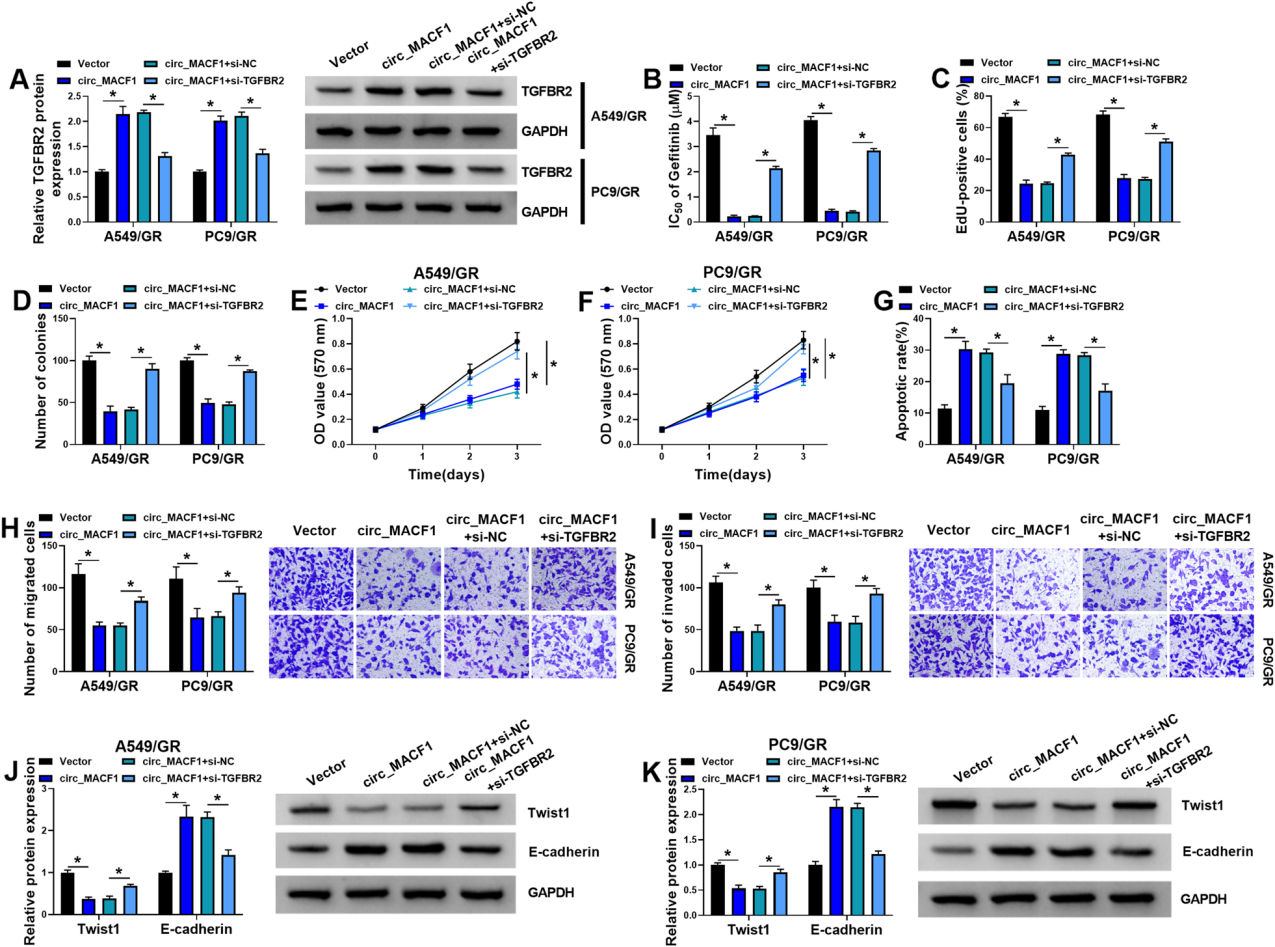
Circ_MACF1 overexpression affects the functional behaviors and gefitinib sensitivity of A549/GR and PC9/GR cells by inducing TGFBR2 expression. A549/GR and PC9/GR cells were transfected with negative control Vector plasmid, circ_MACF1 overexpression plasmid, circ_MACF1 overexpression plasmid + si-NC, or circ_MACF1 overexpression plasmid + si-TGFBR2, followed by the assessment of TGFBR2 protein level by western blot (**A**), IC_50_ value for gefitinib by MTT assay (**B**), cell proliferation by EdU assay (**C**), cell colony formation by colony formation assay (**D**), cell proliferation by MTT assay (**E**, **F**), cell apoptosis by flow cytometry (**G**), cell migration and invasion by transwell assay (**H**, **I**), Twist1 and E-cadherin levels by western blot (**J**, **K**). *P < 0.05

Our above data demonstrated that circ_MACF1 post-transcriptionally regulates TGFBR2 expression through miR-942-5p. We next asked whether TGFBR2 is responsible for the function of circ_MACF1 in A549/GR and PC9/GR cells. To address this, we down-regulated TGFBR2 in circ_MACF1-overexpressing A549/GR and PC9/GR cells (Fig. 7A). Indeed, depletion of TGFBR2 remarkably abrogated circ_MACF1 overexpression-mediated IC_50_ value reduction (Fig. 7B), anti-proliferation, anti-colony formation (Fig. 7C–F), pro-apoptosis (Fig. 7G), anti-migration (Fig. 7H), and anti-invasion (Fig. 7I) effects. Furthermore, TGFBR2 down-regulation attenuated circ_MACF1 overexpression-caused alteration in Twist 1 and E-cadherin expression in A549/GR and PC9/GR cells (Fig. 7J, K). All these findings demonstrate that the effects of circ_MACF1 overexpression depend, at least in part, on the elevation of TGFBR2.

### Overexpression of circ_MACF1 diminishes tumor growth under gefitinib in vivo

**Fig. 8:**
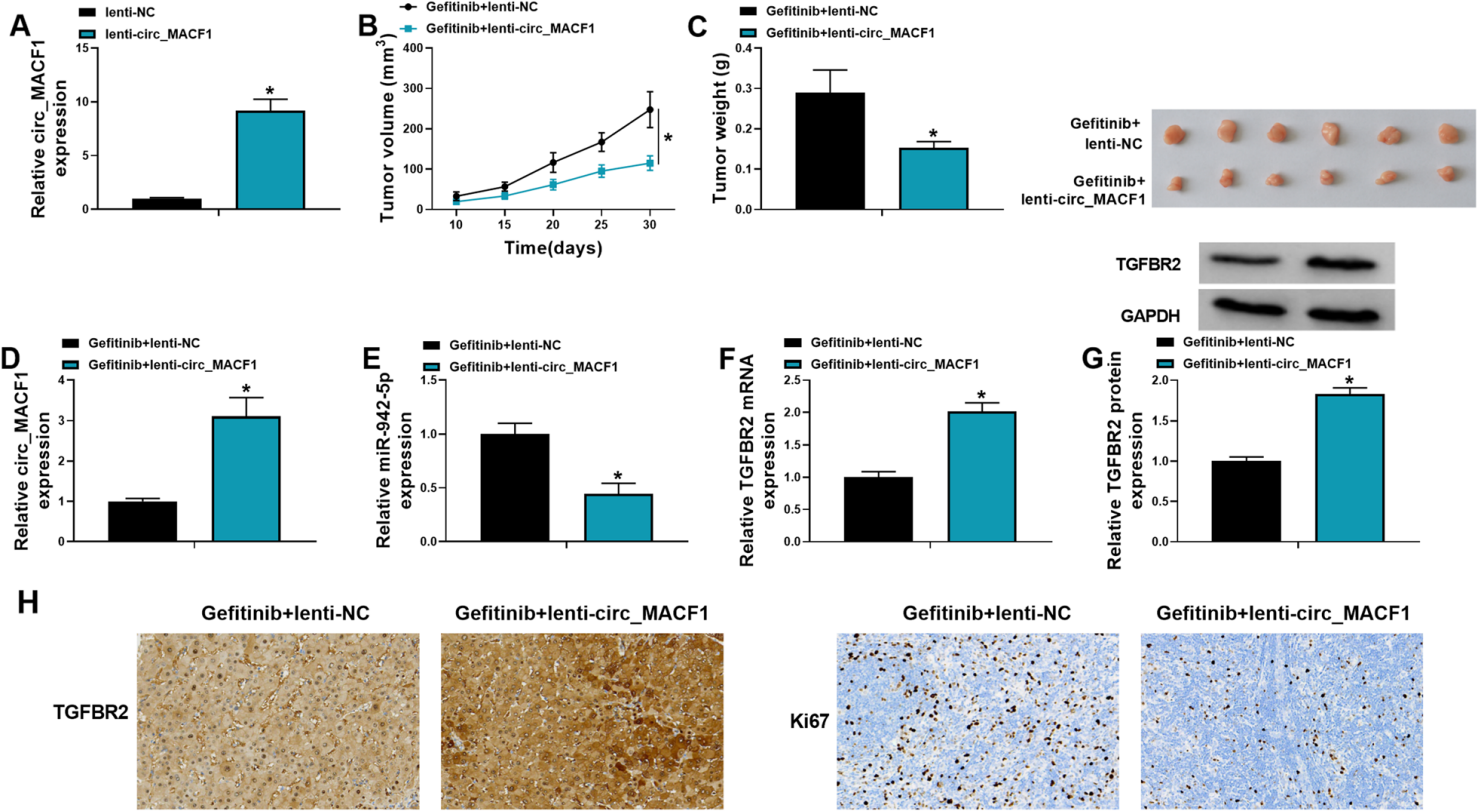
Overexpression of circ_MACF1 inhibits tumor growth under gefitinib in vivo. **A** qRT-PCR analysis of circ_MACF1 expression in A549/GR cells transduced by lentiviruses expressing circ_MACF1 (lenti-circ_MACF1) or a scrambled control (lenti-NC). A549/GR cells transduced by lenti-circ_MACF1 or lenti-NC were implanted into the right flanks of BALB/c nude mice (6 mice per group), with gefitinib administration (30 mg/kg) per os and tumor volume measurement (**B**) 10 days after implantation, and checked for tumor weight and images (**C**), circ_MACF1 expression by qRT-PCR analysis (**D**), miR-942-5p expression by qRT-PCR analysis (**E**), TGFBR2 mRNA level by qRT-PCR analysis (**F**), TGFBR2 protein level by western blot (**G**), TGFBR2 protein level and Ki67 staining by immunohistochemistry (**H**) at the end point (30 days after injection). *P < 0.05

The in vitro studies demonstrated that overexpression of circ_MACF1 causes a growth disadvantage. To further elucidate this observation, we carried out in vivo assays; A549/GR cells transduced by lentiviruses expressing circ_MACF1 (lenti-circ_MACF1) or a scrambled control (lenti-NC) were implanted into the flanks of BALB/c nude mice, with gefitinib administration (30 mg/kg) per os. The effectiveness of lenti-circ_MACF1 transduction in up-regulating circ_MACF1 in A549/GR cells was validated by qRT-PCR (Fig. 8A). Lenti-circ_MACF1-transduced A549/GR cells produced remarkably smaller tumors than the same cells transduced by lenti-NC under gefitinib (Fig. 8B, C). Moreover, lenti-circ_MACF1-transduced tumors exhibited higher levels of circ_MACF1 and TGFBR2 and lower expression of miR-942-5p compared with negative controls under gefitinib (Fig. 8D–H). Additionally, lenti-circ_MACF1-transduced tumors had markedly fewer cells stained for Ki67 staining than the controls under gefitinib (Fig. 8H), reinforcing the repression of tumor growth of circ_MACF1 overexpression. These observations collectively imply that the inhibition of tumor growth under gefitinib may be in part due to overexpression of circ_MACF1 and TGFBR2 and down-regulation of miR-942-5p.

## Discussion

Acquired resistance to gefitinib is one of the primary obstacles for the successful treatment of NSCLC patients with EGFR-activating mutations [6, 20]. To improve the survival of gefitinib-resistant NSCLC, identifying the molecular basis of gefitinib resistance has been challenging. Up to now, circRNAs have been discovered to be implicated in the development of NSCLC cell resistance to various drugs, including gefitinib [12, 13, 21]. Because of the tumor-suppressive role of circ_MACF1 in NSCLC [15], we focused on it in this study.

Human PC9 NSCLC cells harbor a traditional EGFR mutation (exon 19 deletion) and A549 cells express wild-type EGFR, which are the most widely used to investigate gefitinib resistance in NSCLC [22–25]. In this paper, we established two gefitinib-resistant NSCLC cell lines (A549/GR and PC9/GR) to explore the role of circ_MACF1 in resistance mechanisms. Our findings showed a striking down-regulation of circ_MACF1 in A549/GR and PC9/GR cells, suggesting the association between circ_MACF1 expression and gefitinib resistance. Using a series of functional experiments, we first demonstrated that circ_MACF1 is an important regulator in repressing cell malignant behaviors and promoting gefitinib sensitivity of A549/GR and PC9/GR cells. E-cadherin is a marker of epithelial-mesenchymal transition (EMT) and its loss is associated with the enhanced EMT in cancer cells [26]. Twist1, a transcription factor of cell EMT, has been considered as a potential target for overcoming resistance to EGFR-TKIs in EGFR-mutant NSCLC [27]. By checking the alteration of their expression, our findings reinforced the suppressive function of circ_MACF1 in gefitinib resistance. Additionally, as reported for other circRNAs [28, 29], circ_MACF1 is RNase R resistance because the 3’ and 5’ ends are covalently linked giving rise to circular molecules [7].

Numerous studies have documented the oncogenic activity of miR-942-5p in human cancers, such as melanoma, hepatocellular carcinoma, and colorectal cancer [30–32]. Up-regulated miR-942-5p (also called miR-942) in NSCLC serum may be a potential biomarker for NSCLC diagnosis and prognosis [33]. Moreover, miR-942-5p is capable of promoting NSCLC progression by regulating its targeted mRNAs [34, 35]. Here, we first ascertained that circ_MACF1 directly targets miR-942-5p and suppresses its expression. Furthermore, we found that miR-942-5p is a downstream mediator of circ_MACF1 in affecting the functional behaviors and gefitinib sensitivity of A549/GR and PC9/GR cells. Dong et al*.* demonstrated that circFBXW7 suppressed the progression of lung adenocarcinoma through sponging miR-942-5p [36], which prompts that it is possible that multiple circRNAs are responsible for the regulation of miR-942-5p in NSCLC by combining our findings. Additionally, Wang et al. uncovered that long non-coding RNA (lncRNA) LIFR-AS1 worked as an anti-tumor factor in NSCLC by inhibiting miR-942-5p activity [35]. Whether the circFBXW7/miR-942-5p and LIFR-AS1/miR-942-5p axes are involved in the gefitinib resistance of NSCLC needs to be further explored.

TGFBR2, a key member of the TGF-β pathway, is frequently deleted during carcinogenesis in many types of cancers, including NSCLC [37, 38]. TGFBR2 has been shown as a potent tumor inhibitor in NSCLC [39–41]. Moreover, TGFBR2 is associated with drug resistance of NSCLC [42, 43]. In this report, our findings first uncovered that TGFBR2 is directly targeted and regulated by miR-942-5p, and TGFBR2 overexpression phenocopied miR-942-5p depletion in inhibiting the malignant behaviors and promoting gefitinib sensitivity of A549/GR and PC9/GR cells. Previous work also reported that miR-942-5p contributed to NSCLC development by inducing epithelial-mesenchymal transition (EMT) through targeting BARX2 [34]. However, whether the miR-942-5p/BARX2 axis is involved in the development of gefitinib resistance remains to be elucidated. More importantly, we first established the post-transcriptional regulation of circ_MACF1 on TGFBR2 expression through miR-942-5p. Similarly, Liu and colleagues ascertained that circ_MACF1 sponged miR-544a to suppress NSCLC progression by post-transcriptionally regulating the expression of FBXW7 [15], wherein FBXW7 plays an important role in gefitibin resistance in NSCLC [44]. There may be other mechanisms that remain to be elucidated in the regulation of circ_MACF1.

The in vivo experiments implied that tumor growth under gefitinib may be regulated by the circ_MACF1/miR-942-5p/TGFBR2 axis. However, further investigations should be carried out in further work. Additionally, these findings of clinical data from 11 NSCLC patients harboring EGFR-activating mutations suggested that abnormal expression of circ_MACF1 and miR-942-5p may be associated with gefitinib resistance. Restricted by the small sample size, future studies are required to expand the analysis of their expression in plasma to a larger cohort of patients to validate that altered levels of circ_MACF1 and miR-942-5p correlate with gefitinib resistance of NSCLC.

In summary, our current observations demonstrated circ_MACF1 as an important regulator in suppressing cell malignant behaviors and promoting gefitinib sensitivity of A549/GR and PC9/GR cells by targeting miR-942-5p to induce TGFBR2 expression. Our study identified a novel complex circuitry underlying gefitinib resistance, underscoring the potential diagnostic and therapeutic implications.

## Supplementary Information

**Additional file 1: Figure S1**. The Selection of circ_MACF1, miR-942-5p and TGFBR2. (A) Expression of circRNAs in A549 and A549/GR cells by qRT-PCR analysis. (B and C) RNA pull-down assays showing the level of circ_MACF1 and the enrichment levels of miRNAs in A549/GR cells transfected with or without vector or circ_MACF1 expressing plasmid. (D) Expression of mRNA levels in A549/GR cells transfected with anti-miR-NC or anti-miR-942-5p by qRT-PCR analysis. *P < 0.05.

**Additional file 2: Figure S2**. The Circ_MACF1 was underexpressed and miR-942-5p was overexpressed in gefitinib-resistant NSCLC plasma. qRT-PCR analysis of circ_MACF1 (A) and miR-942-5p (B) in 11 pairs of pre- (Tumor-responsive) and post-gefitinib (Tumor-resistant) treated plasma samples. *P < 0.05.

**Additional file 3: Table S1**. The Sequences of qRT-PCR primers and oligonucleotides.

## Data Availability

Due to the nature of this research, participants of this study did not agree for their data to be shared publicly, so supporting data is not available.

## Abbreviations

NSCLC: Non-small cell lung cancer
EGFR: Epidermal growth factor receptor
circ_MACF1: Microtubule actin crosslinking factor 1
TGFBR2: Transforming growth factor beta receptor 2
