# Supplementary figures and images for: A novel circ_MACF1/miR-942-5p/TGFBR2 axis regulates the functional behaviors and drug sensitivity in gefitinib-resistant non-small cell lung cancer cells
Source: BMC Pulm Med. 2022 Jan 7;22:27. doi: 10.1186/s12890-021-01731-z (PMC8742390; doi:10.1186/s12890-021-01731-z)

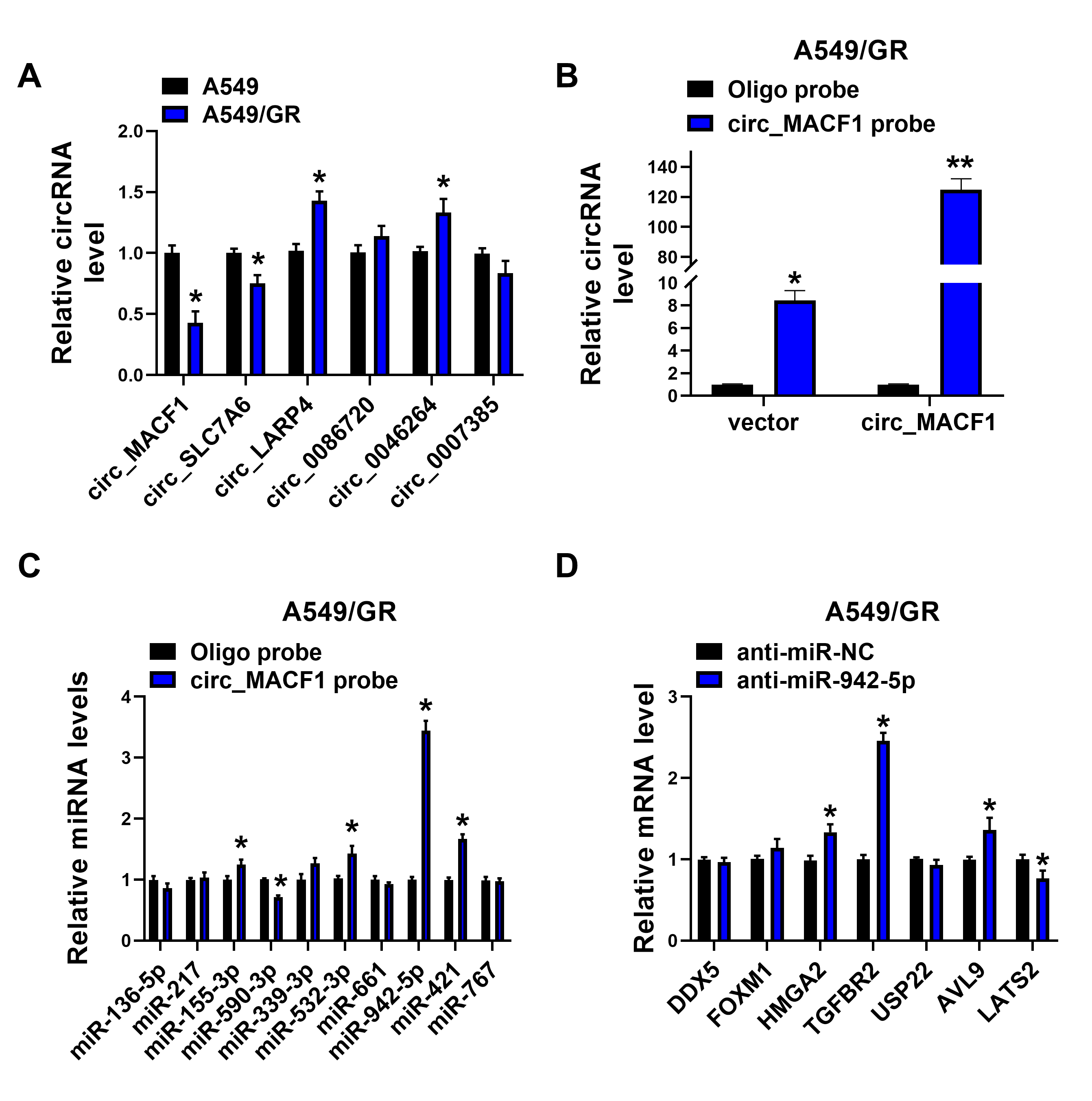

Supplement: Supplementary file 1 — Additional file 1: Figure S1. The Selection of circ_MACF1, miR-942-5p and TGFBR2. (A) Expression of circRNAs in A549 and A549/GR cells by qRT-PCR analysis. (B and C) RNA pull-down assays showing the level of circ_MACF1 and the enrichment levels of miRNAs in A549/GR cells transfected with or without vector or circ_MACF1 expressing plasmid. (D) Expression of mRNA levels in A549/GR cells transfected with anti-miR-NC or anti-miR-942-5p by qRT-PCR analysis. *P < 0.05. [file 12890_2021_1731_MOESM1_ESM.tif]

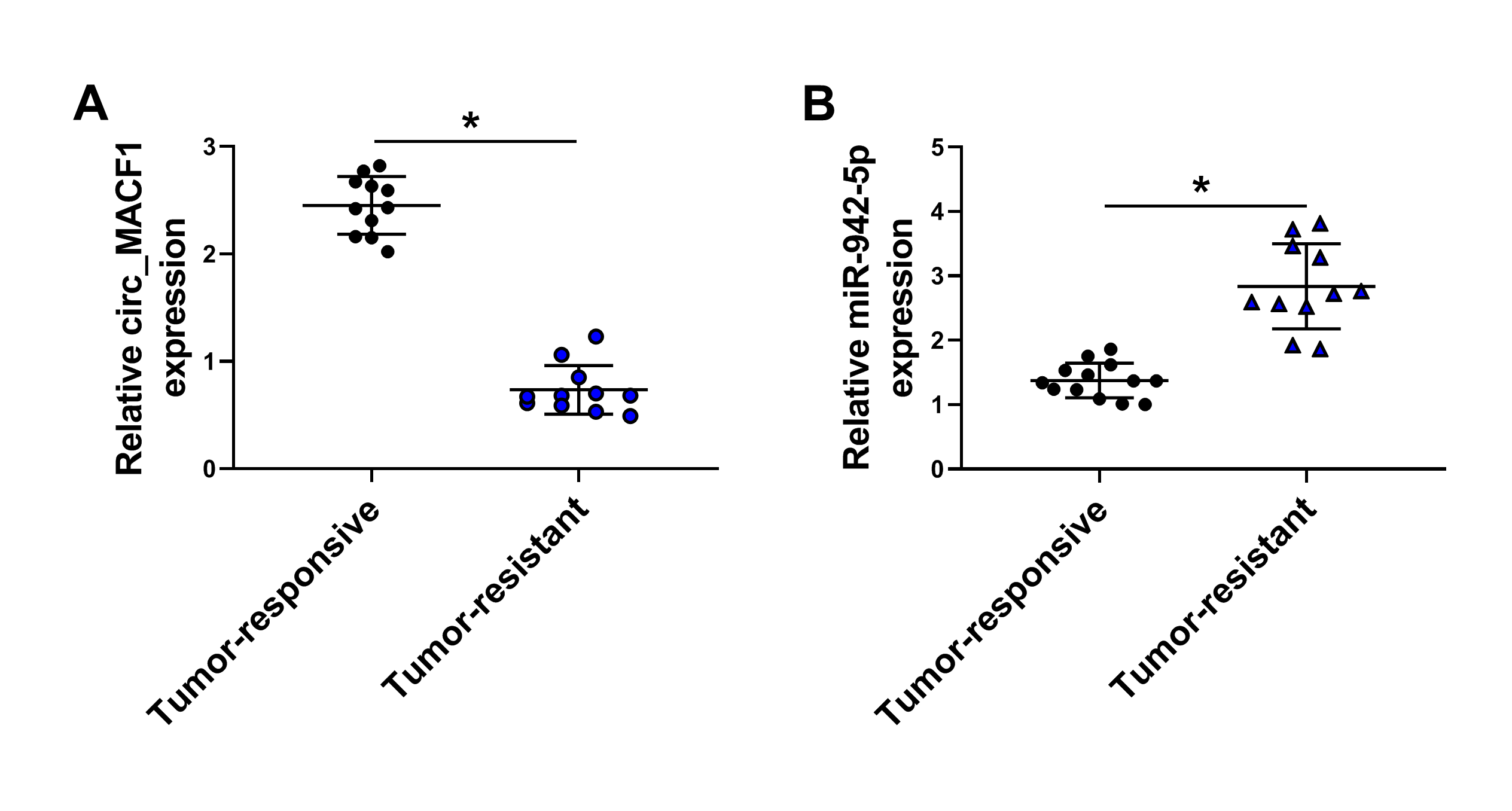

Supplement: Supplementary file 2 — Additional file 2: Figure S2. The Circ_MACF1 was underexpressed and miR-942-5p was overexpressed in gefitinib-resistant NSCLC plasma. qRT-PCR analysis of circ_MACF1 (A) and miR-942-5p (B) in 11 pairs of pre- (Tumor-responsive) and post-gefitinib (Tumor-resistant) treated plasma samples. *P < 0.05. [file 12890_2021_1731_MOESM2_ESM.tif]
