# Supplementary material for: A novel circ_MACF1/miR-942-5p/TGFBR2 axis regulates the functional behaviors and drug sensitivity in gefitinib-resistant non-small cell lung cancer cells
Source: BMC Pulm Med. 2022 Jan 7;22:27. doi: 10.1186/s12890-021-01731-z (PMC8742390; doi:10.1186/s12890-021-01731-z)
Supplement: Supplementary file 3 — Additional file 3: Table S1. The Sequences of qRT-PCR primers and oligonucleotides. [file 12890_2021_1731_MOESM3_ESM.docx]

**Supplement Table 1. Sequences of qRT-PCR primers and oligonucleotides**

| Sequence (5’-3’) | | |
| --- | --- | --- |
| circ_MACF1 | Forward | CCTTTGTGATGGTTCTGCAA |
|  | Reverse | GATCCAGGGAATGAGGGAGT |
| MACF1 mRNA | Forward  Reverse | GAAACTGGAGCGAGCAGAGT  TATGCAGGCTCTGAAGGTGC |
| TGFBR2  GAPDH | Forward  Reverse  Forward  Reverse | GCTCCCAGCCTTCATCCTTT  TTGAGCAATCAGGAGCCCAG  GACAGTCAGCCGCATCTTCT  GCGCCCAATACGACCAAATC |
| miR-942-5p | Forward | GCCGAGTCTTCTCTGTTTTGG |
|  | Reverse | CTCAACTGGTGTCGTGGA |
| β-actin | Forward | CTCGCCTTTGCCGATCC |
|  | Reverse | GGGGTACTTCAGGGTGAGGA |
| U6 | Forward | CTCGCTTCGGCAGCACA |
|  | Reverse | AACGCTTCACGAATTTGCGT |
| si-NC | AACAGUCGCGUUUGCGACUGG | |
| si-TGFBR2 | UAUUCAUAUUUAUAUACAGGC | |
| miR-NC mimic | CGAUCGCAUCAGCAUCGAUUGC | |
| miR-942-5p mimic | UCUUCUCUGUUUUGGCCAUGUG | |
| anti-miR-NC | CAGUACUUUUGUGUAGUACAA | |
| anti-miR-942-5p | CACAUGGCCAAAACAGAGAAGA | |
